# Applications of Cattaneo–Christov fluxes on modelling the boundary value problem of Prandtl fluid comprising variable properties

**DOI:** 10.1038/s41598-021-97420-2

**Published:** 2021-09-08

**Authors:** Umar Nazir, Muhammad Sohail, Umair Ali, El-Sayed M. Sherif, Choonkil Park, Jung Rye Lee, Mahmoud M. Selim, Phatiphat Thounthong

**Affiliations:** 1 Department of Applied Mathematics and Statistics, Institute of Space Technology, P.O. Box 2750, Islamabad, 44000 Pakistan; 2 Mechanical Engineering Department, College of Engineering, King Saud University, P.O. Box 800, Al-Riyadh, 11421 Saudi Arabia; 3 Research Institute for Natural Sciences, Hanyang University, Seoul, 04763 Korea; 4 Department of Data Science, Daejin University, Pocheon, Kyunggi 11159 Korea; 5 Department of Mathematics, Al-Aflaj College of Science and Humanities Studies, Prince Sattam Bin Abdulaziz University, Al-Aflaj, 710-11912 Saudi Arabia; 6 Department of Mathematics, Suez Faculty of Science, Suez University, Suez, 34891 Egypt; 7 Department of Teacher Training in Electrical Engineering, Faculty of Technical Education, Renewable Energy Research Centre, King Mongkut’s University of Technology North Bangkok, 1518 Pracharat 1 Road, Bangsue, Bangkok, 10800 Thailand

**Keywords:** Applied mathematics, Computational science, Mathematics and computing, Nanoscience and technology

## Abstract

Stretched flows have numerous applications in different industrial, biomedical and engineering processes. Current research is conducted to examine the flow phenomenon of Prandtl fluid model over a moveable surface. The phenomenon of mass and thermal transportation is based on generalized theory of Cattaneo–Christov which considers the involvement of relaxation times. In addition to these, variable characteristics of thermal conductivity and diffusion coefficient are considered as a function of temperature. The physical problem in Cartesian coordinate system is modeled via boundary layer theory which yields a coupled system of partial differential equations. Group scaling transportation is applied to model these PDEs system. The converted equations have been approximated via optimal homotopic scheme. The efficiency and validity of used approach has been shown by computing the error analysis and establishing a comparative study. It is noted that the enhancement in magnetic parameter plays a controlling role for velocity field and it augment the concentration and temperature fields. Furthermore, increase in thermal relaxation parameter and Prandtl number maintains the fluid temperature.

## Introduction

The rheology of non-Newtonian fluids has complex nature and it cannot be exactly described by the stress–strain relatively as proposed by Newton. Non-Newtonian materials appear frequently, and it has applications in different mechanisms. One of the important non-Newtonian fluid is Prandtl fluid model^1^ which is describes the following constitutive relation

$$ \tau = \left[ {A\left( {\frac{\partial v}{{\partial y}}} \right)^{2} + A\left( {\frac{\partial u}{{\partial y}}} \right)^{2} } \right]^{{ - \frac{1}{2}}} \sin^{ - 1} \left[ {\frac{1}{C}\left[ {\left( {\frac{\partial u}{{\partial y}}} \right)^{2} + \left( {\frac{\partial v}{{\partial y}}} \right)^{2} } \right]^{\frac{1}{2}} \frac{\partial u}{{\partial y}}} \right] $$

where “$\tau$” denotes the stress tensor for Prandtl model, ““$A$ and $C$”” are material parameters. The classification of fluids (non-Newtonian) is based on their behaviors and shear thinning category of fluids is considered as important in fluids. The model related to Prandtl fluid is known as a shear thinning liquid. Human blood, paint, blood, industrial and polymers products are examples of Prandtl fluid. This kind of liquid performs like a non-Newtonian fluid. Several applications of such non-Newtonion fluid in sugar production, cement industry, making shampoos and drilling muds etc. Several researchers have worked on this model by considering the different physical effects under different circumstances and assumptions. For instance, Hamid et al.^1^ worked on unsteady natural convection flow of Prandtl model over a moveable surface. They considered the chemical reaction, radiation and magnetohydrodynamic influences. They studied the mass and thermal transport on Prandtl model by applying the boundary layer theory on the associated conservation laws. They obtained the solution via numerical method namely “Crank-Nicolson” coded in MATLAB symbolic computational package. They recorded that augmenting volume of unsteadiness parameter upsurges the momentum, thermal and concentration fields. Rajesh and Rajasekhara Gowd^2^ discussed the rheology of Prandtl fluid during the transport of solute particles and thermal energy past a porous vertical channel. They also considered thermal radiation phenomena along with magnetic field. Later on, the governing model is transformed into PDEs by appropriate substitution and then solved analytically. They found a rise in velocity field against gravitational parameters. Eldabe et al.^3^ focused on peristaltic motion of Prandtl liquid considering chemical reaction and electrical conductivity (variable) obeying the mixed convection in symmetric channel. They used differential transform procedure to tackle the resulting equation. They illustrated the solutions through graphs. They noticed the decline in pressure profile against chemical reaction parameters. Flow of Prandtl model over a rotating slippery sheet with convective condition, radiation and concentration dependent diffusion coefficient was investigated by Sajid et al.^4^ They recorded the amplification in thermal profile for radiation parameter and decline in velocity field against rotating parameter. Reddy et al.^5^ modeled the compartment of modified heat flux. They handled the resulting equations as a numerically. They reported that velocity profile escalates against elastics and Prandtl fluid parameters Transient numerical solution for Prandtl model in rotating surface was discussed by Le et al.^6^. They reported the phenomenon of bifurcation. Transportation of heat energy as well as mass has significant application in different field of applied sciences. Ahmad et al.^7^ used non-Fourier’s model to study the heating effects on micropolar fluid over a hot surface. They assumed the slip conditions, thermal radiation and magneto-hydrodynamic effect in their research. Numerically they handled the resulting modeled expression. They studied the entropy analysis and recorded that mounting value of material parameter and Reynolds number entrances the entropy generation. Tassadiq^8^ studied the homotopic solution for micropolar hybrid nanofluid model with dissipation and Joule heating effects and they solved the modeled equations with the help of numerical approach. Through graphical solution, they predicted the decline in velocity filed against Hartmann number and micropolar parameters. Ahmad et al.^9^ reported the phenomenon of shallow wave dispersive equation arising in mathematical physics. They presented the graphical analysis of two methods. Tulu and Ibrahim^10^ studied the use of CNT-Ethylene Glycol to enhance the thermal transport over a rotating stretchable disc with modified heat flux model. They solved the nonlinear transport equation numerically. Through graphs they recorded the behavior of solution against numerous emerging parameters. They noticed that radial stretching of the disc improves the cooling effect which is beneficial to control the thermal stability of the system. Numerical solution of Jeffrey liquid model is captured by Mabood et al.^11^ with heat transport phenomenon. Graphically they plotted the behavior of parameters for the assisting and opposing flow situations. They mentioned the decline in temperature field against Deborah number. Finite element analysis is applied to study the double diffusion theories on Casson model by Ali et al.^12^. They noticed the decrease in primary and secondary velocity against magnetic parameter and fluid parameter, where, rise in concentration and thermal profile is seen. Khan et al.^13^ presented the theoretical analysis on thermal transport with the inclusion of different nanoparticles. They noticed the depreciation in thermal field for Prandtl number. Heat transport in Oldroyd-B model over elastic surface in inspected by Ramana et al.^14^ numerically. Jakeer et al.^15^ considered finite volume approach to study the thermal transport in lid-driven porous cavity. They plotted the streamlines against flow controlling parameters. Nadeem et al.^16^ modeled the transport phenomenon of thermal energy in Newtonian liquid in the attendance of non-Fourier’s theory. They investigated this transport phenomenon past heated surface via Optimal HAM approach. Ahmad et al.^17^ scrutinized theory related to the non-Fourier in micropolar fluid considering the presence of heat absorption. They used the role nanoparticles in micropolar fluid and they also simulated the entropy generation mechanism. Ahmad et al.^18^ simulated the features of thermal energy and solute particles in micropolar involvement of nanoparticles and hybrid nanoparticles in base fluid called water. They used the stretching heated surface for measurement of considering features in the presence of non-Fourier’s theory along with activation energy. Ahmad and Nadeem^19^ analyzed the role of non-Fourier’s law in the energy equation along with slip conditions (Thomson and Troian). Several recently studied contribution are reported in^20–26^ and references therein.

Available literature tells that no study is conducted with variable thermo-physical properties for 3D Prandtl model with double diffusion theories. This model covers all aspects which are missing in published work. The adjustment of present model is considered into following: Literature with detailed description is performed in “Introduction” section, fluid rheology with heat and mass transport phenomenon are listed in “Mathematical formulation of transport problem” section with dimensionless equations, “Numerical method for solution” section contains the methodology, “Discussion and analysis on graphical and tabular results” and “Concluding key points” sections describe the detailed discussion on graphical outcomes with conclusion.

## Mathematical formulation of transport problem

**Figure 1:**
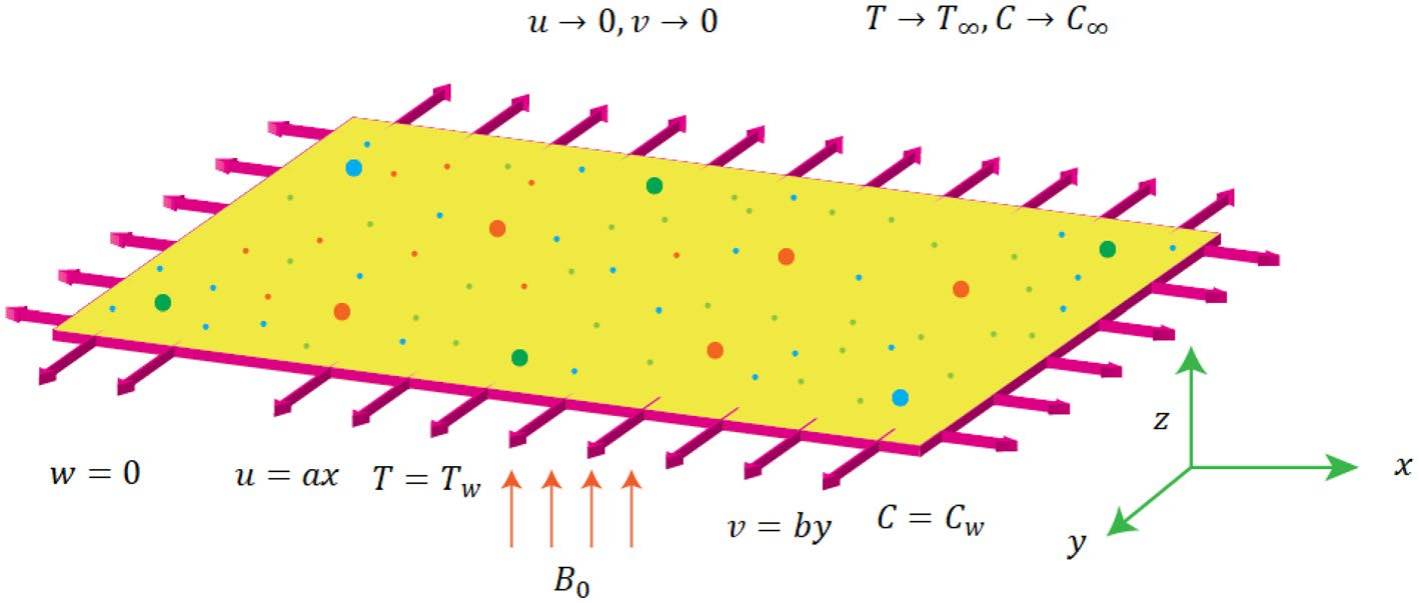
Flow diagram of Prandtl fluid.

Steady incompressible flow of Prandtl fluid heat and mass transport is considered in this project as shown in Fig. 1. The stretching of sheet is responsible to maintain the flow. The sheet is stretched in *x*- and *y*- direction respectively and flow region is $z \ge 0.$ The direction of uniform magnetic field is considered to normal of *x*- and *y*- directions as shown in Fig. 1. The characteristics of heat energy and diffusion of fluid particles are reported under the action of timrelaxations. The concept fluxes (heat and mass) including relaxation times are

1 $$ {\varvec{Q}} + \alpha_{1} \left[ {\frac{{\partial {\varvec{Q}}}}{\partial t} + {\varvec{V}} \cdot \nabla {\varvec{Q}} - {\varvec{Q}} \cdot \nabla {\mathbf{V}} + \left( {\nabla \cdot {\mathbf{V}}} \right){\mathbf{Q}}} \right] = - K\left( T \right)\nabla {\text{T}}, $$

2 $$ {\varvec{J}} + \alpha_{2} \left[ {\frac{{\partial {\varvec{J}}}}{\partial t} + {\varvec{V}} \cdot \nabla {\mathbf{J}} - {\mathbf{J}} \cdot \nabla {\mathbf{V}} + \left( {\nabla \cdot {\mathbf{V}}} \right){\mathbf{J}}} \right] = - D\left( T \right)\nabla {\mathbf{J}}. $$

Using incompressibility and steady flow assumptions, we get

3 $$ {\varvec{Q}} + \alpha_{1} \left[ {{\varvec{V}} \cdot \nabla {\mathbf{Q}} - {\mathbf{Q}} \cdot \nabla {\mathbf{V}}} \right] = - K\left( T \right)\nabla {\text{T,}} $$

4 $$ {\varvec{J}} + \alpha_{2} \left[ {{\varvec{V}} \cdot \nabla {\mathbf{J}} - {\mathbf{J}} \cdot \nabla {\mathbf{V}}} \right] = - D\left( T \right)\nabla {\mathbf{J}}. $$

where “${\varvec{Q}}$” is heat flux, “${\varvec{V}}$” represents velocity field, “$K\left( T \right)$” the thermal conductivity, “${\varvec{J}}$” the mass flux, “D(T)” the diffusion coefficient, “$\alpha_{1}$” and “$\alpha_{2}$” are thermal and concentration relaxation times using the boundary layer theory the conservation laws for Prandtl model takes the form

5 $$ \frac{\partial v}{{\partial y}} + \frac{\partial w}{{\partial z}} + \frac{\partial u}{{\partial x}} = 0,{ } $$

6 $$ \left( {v\frac{\partial }{\partial y} + w\frac{\partial }{\partial z} + u\frac{\partial }{\partial x}} \right)u = \frac{A}{C}\delta \frac{{\partial^{2} u}}{{\partial z^{2} }} + \delta \frac{A}{{2C^{3} }}\left( {\frac{\partial u}{{\partial z}}} \right)^{2} \frac{{\partial^{2} u}}{{\partial z^{2} }}{ } - { }\frac{ \sigma }{\rho }uB_{0}^{2} , $$

7 $$ \left( {v\frac{\partial }{\partial y} + w\frac{\partial }{\partial z} + u\frac{\partial }{\partial x}} \right)v = \frac{A}{C}\delta \frac{{\partial^{2} v}}{{\partial z^{2} }} + \delta \frac{A}{{2C^{3} }}\left( {\frac{\partial v}{{\partial z}}} \right)^{2} \frac{{\partial^{2} v}}{{\partial z^{2} }}{ } - { }\frac{{ \sigma vB_{0}^{2} }}{\rho } ,{ } $$

8 $$ \begin{aligned} & uT_{x} + vT_{y} + wT_{z} + \alpha_{1} \left[ {\begin{array}{*{20}c} {\left( {v\frac{\partial }{\partial y} + w\frac{\partial }{\partial z} + u\frac{\partial }{\partial x}} \right)uT_{x} + \left( {v\frac{\partial }{\partial y} + w\frac{\partial }{\partial z} + u\frac{\partial }{\partial x}} \right)vT_{y} } \\ { + \left( {v\frac{\partial }{\partial y} + w\frac{\partial }{\partial z} + u\frac{\partial }{\partial x}} \right)wT_{z} + 2uvT_{xy} + 2vwT_{yz} } \\ { + 2uwT_{xz} + u^{2} T_{xx} + v^{2} T_{yy} + w^{2} T_{zz} } \\ \end{array} } \right] \\ & = \nabla \left[ {K\left( T \right)\nabla {\text{T}}} \right], \\ \end{aligned} $$

9 $$ \begin{aligned} & uC_{x} + vC_{y} + wC_{z} + \alpha_{2} \left[ {\begin{array}{*{20}c} {\left( {v\frac{\partial }{\partial y} + w\frac{\partial }{\partial z} + u\frac{\partial }{\partial x}} \right)uC_{x} + \left( {v\frac{\partial }{\partial y} + w\frac{\partial }{\partial z} + u\frac{\partial }{\partial x}} \right)vC_{y} } \\ { + \left( {v\frac{\partial }{\partial y} + w\frac{\partial }{\partial z} + u\frac{\partial }{\partial x}} \right)wC_{z} + 2uvC_{xy} + 2vwC_{yz} } \\ { + 2uwC_{xz} + u^{2} C_{xx} + v^{2} C_{yy} + w^{2} C_{zz} } \\ \end{array} } \right] \\ & = \nabla \left[ {{\text{D}}\left( {\text{T}} \right)\nabla {\text{C}}} \right]. \\ \end{aligned} $$

Boundary conditions (BCs) are necessary to compute the solution of derived problem and BCs of present model are

10 $$ \left\{ {\begin{array}{*{20}l} {u = U_{w} = ax, v = V_{w} = by,w = 0, T = T_{w} , C = C_{w} \;at\; z = 0.} \hfill \\ {u \to 0, v \to 0, T \to T_{\infty } , C \to C_{\infty } \;for\; z \to \infty . } \hfill \\ \end{array} } \right. $$

With the use of following similarity variables governing laws with associated conditions reduces to

11 $$ \left\{ {\begin{array}{*{20}l} {u = axf^{\prime}\left( \xi \right), v = ayg^{\prime}\left( \xi \right),w = - \left( {a\delta } \right)^{\frac{1}{2}} \left[ {f\left( \xi \right) + g\left( \xi \right)} \right], } \hfill \\ {\theta \left( \xi \right) = \frac{{T - T_{\infty } }}{{T_{w} - T_{\infty } }}, \phi \left( \xi \right) = \frac{{C - C_{\infty } }}{{C_{w} - C_{\infty } }}, \xi = \left( {\frac{{\text{a}}}{\delta }} \right)^{\frac{1}{2}} z.} \hfill \\ \end{array} } \right. $$

12 $$ \beta_{1} f^{\prime\prime\prime}\left( \xi \right) + \left[ {f\left( \xi \right) + g\left( \xi \right)} \right]f^{\prime\prime}\left( \xi \right) - \left[ {f^{\prime}\left( \xi \right)} \right]^{2} + \beta_{2} f^{\prime\prime\prime}\left( \xi \right)\left[ {f^{\prime\prime}\left( \xi \right)} \right]^{2} - Mf^{\prime}\left( \xi \right) = 0, $$

13 $$ \beta_{1} g^{\prime\prime\prime}\left( \xi \right) + \left[ {f\left( \xi \right) + g\left( \xi \right)} \right]g^{\prime\prime}\left( \xi \right) - \left[ {g^{\prime}\left( \xi \right)} \right]^{2} + \beta_{2} g^{\prime\prime\prime}\left( \xi \right)\left[ {g^{\prime\prime}\left( \xi \right)} \right]^{2} - Mg^{\prime}\left( \xi \right) = 0, $$

14 $$ \begin{aligned} & \frac{1}{Pr}\left[ {1 + \epsilon_{1} \theta \left( \xi \right)} \right]\theta^{\prime\prime}\left( \xi \right) + \left[ {f\left( \xi \right) + g\left( \xi \right)} \right]\theta^{\prime}\left( \xi \right) \\ & \quad - \delta_{1} \left[ {f\left( \xi \right) + g\left( \xi \right)\left( {f^{\prime}\left( \xi \right) + g^{\prime}\left( \xi \right)} \right)\theta^{\prime}\left( \xi \right) + \left( {f\left( \xi \right) + g\left( \xi \right)} \right)^{2} \theta^{\prime\prime}\left( \xi \right)} \right] = 0, \\ \end{aligned} $$

15 $$ \begin{aligned} & \frac{1}{Sc}\left[ {1 + \epsilon_{2} \theta \left( \xi \right)} \right]\phi^{\prime\prime}\left( \xi \right) + \left[ {f\left( \xi \right) + g\left( \xi \right)} \right]\phi^{\prime}\left( \xi \right) \\ & \quad - \delta_{2} \left[ {f\left( \xi \right) + g\left( \xi \right)\left( {f^{\prime}\left( \xi \right) + g^{\prime}\left( \xi \right)} \right)\phi^{\prime}\left( \xi \right) + \left( {f\left( \xi \right) + g\left( \xi \right)} \right)^{2} \phi^{\prime\prime}\left( \xi \right)} \right] = 0, \\ \end{aligned} $$

16 $$ \left\{ {\begin{array}{*{20}l} {\xi = 0, g = f = 0, f^{\prime} = 1, g^{\prime} = \alpha , \theta = \phi = 1,} \hfill \\ { \xi = \infty , f^{\prime} = g^{\prime} = 0, \theta = \phi = 0. } \hfill \\ \end{array} } \right. $$

where “$\beta_{1}$” presents Prandtl fluid parameter, “$\beta_{2}$” the elastic parameter, “$\alpha$” ratio parameter, “*M*” the magnetic parameter, “*Pr*” the Prandtl number, “*Sc*” the Schmidt number, “$\epsilon_{1}$ and $\epsilon_{2}$” are small parameters and “$\delta_{1}$ and $\delta_{2}$” denotes thermal and concentration relaxation times.

### Physical quantities

To understand the several practical applications, the study of stress, heat and mass transfer rate have significant use. The empirical relations of these quantities are

$$ C_{fx} = \frac{{\tau_{zx} }}{{\rho \left( U \right)^{2} }}, C_{fy} = \frac{{\tau_{zy} }}{{\rho \left( {V_{w} } \right)^{2} }}, $$

17 $$ Nu_{x} = - \frac{{xQ_{w} }}{{K\left( T \right)\left( {T_{w} - T_{\infty } } \right)}}, Sh_{x} = - \frac{{xJ_{w} }}{{D\left( T \right)\left( {C_{w} - C_{\infty } } \right)}},U_{w} = ax, V = by, $$

where $$\tau_{zx} = \frac{A}{C}u_{z} + \frac{A}{{6C^{3} }}\left( {u_{z} } \right)^{3} \left. \right|_{z = 0} ,$$ $$\tau_{zy} = \frac{A}{C}v_{z} + \frac{A}{{6C^{3} }}\left( {v_{z} } \right)^{3} \left. \right|_{z = 0} ,$$ $$Q_{w} = - K\left( T \right)\nabla T, $$

$$ J_{w} = - D\left( T \right)\nabla {\text{C}}, $$

the dimensionless form is

18 $$ C_{fx} = \beta_{1} f^{\prime\prime\prime}\left( \xi \right) + \beta_{2} \left[ {f^{\prime\prime\prime}\left( \xi \right)} \right]^{2} \left. \right|_{\xi = 0} ,\;C_{fy} = \beta_{1} g^{\prime\prime\prime}\left( \xi \right) + \beta_{2} \left[ {g^{\prime\prime\prime}\left( \xi \right)} \right]^{2} \left. \right|_{\xi = 0} , $$

19 $$ Hu_{x} = - \left[ {1 + \epsilon_{1} \theta \left( \xi \right)} \right]\theta^{\prime}\left( 0 \right),\;Lh_{x} = - \left[ {1 + \epsilon_{2} \theta \left( \xi \right)} \right]\phi^{\prime}\left( 0 \right). $$

## Numerical method for solution

In this section, comprehensive literature survey on the considered topic is presented and “Mathematical formulation of transport problem” section comprises the mathematical form of the considered model-along with associated conditions. The current approach has ability to simulate complex flow problems. Several analytical and numerical schemes exist for the solution of differential equations. Here, optimal homotopy scheme is proposed for the solution. This procedure requires the linear operators and initial guess for the start of algorithm. These are

20 $$ \left\{ {\begin{array}{*{20}l} {L_{F} = \frac{{D^{3} }}{{D\xi^{3} }} - \frac{D}{D\xi }, L_{G} = \frac{{D^{3} }}{{D\xi^{3} }} - \frac{D}{D\xi }, } \hfill \\ {L_{T} = \frac{{D^{2} }}{{D\xi^{2} }} - 1, L_{C} = \frac{{D^{2} }}{{D\xi^{2} }} - 1, } \hfill \\ \end{array} } \right. $$

21 $$ \left\{ {\begin{array}{*{20}l} {f_{a} \left( \xi \right) = 1 - e^{ - \xi } , g_{a} = \alpha \left[ {1 - e^{ - \xi } } \right], } \hfill \\ {\theta_{a} \left( \xi \right) = e^{ - \xi } , \phi_{a} \left( \xi \right) = e^{ - \xi } , } \hfill \\ \end{array} } \right. $$

The operators in Eq. (20) obey

$$ L_{F} \left[ {\beta_{1} + \beta_{2} e^{ - \xi } + \beta_{3} e^{\xi } } \right] = 0, $$

$$ L_{G} \left[ {\beta_{4} + \beta_{5} e^{ - \xi } + \beta_{6} e^{\xi } } \right] = 0, $$

$$ L_{T} \left[ {\beta_{7} e^{ - \xi } + \beta_{8} e^{\xi } } \right] = 0, $$

$$ L_{C} \left[ {\beta_{9} e^{\xi } + \beta_{10} e^{ - \xi } } \right] = 0, $$

where $\beta_{m} \left( {m = 1,2, \ldots ,10} \right)$ are unknowns.

Using the concepts of minimization of average squared residual error^21^

$$ e_{m}^{f} = \frac{1}{i + 1}\mathop \sum \limits_{r = 0}^{i} \left[ {M_{f} \left( {\mathop \sum \limits_{l = 0}^{m} \hat{f}\left( \xi \right), \mathop \sum \limits_{l = 0}^{m} \hat{g}\left( \xi \right)} \right)} \right]^{2} , $$

$$ e_{m}^{g} = \frac{1}{i + 1}\mathop \sum \limits_{r = 0}^{i} \left[ {M_{g} \left( {\mathop \sum \limits_{l = 0}^{m} \hat{f}\left( \xi \right), \mathop \sum \limits_{l = 0}^{m} \hat{g}\left( \xi \right)} \right)} \right]^{2} , $$

$$ e_{m}^{\theta } = \frac{1}{i + 1}\mathop \sum \limits_{r = 0}^{i} \left[ {M_{\theta } \left( {\mathop \sum \limits_{l = 0}^{m} \hat{f}\left( \xi \right), \mathop \sum \limits_{l = 0}^{m} \hat{g}\left( \xi \right), \mathop \sum \limits_{l = 0}^{m} \hat{\theta }\left( \xi \right)} \right)} \right]^{2} , $$

$$ e_{m}^{\phi } = \frac{1}{i + 1}\mathop \sum \limits_{r = 0}^{i} \left[ {M_{\phi } \left( {\mathop \sum \limits_{l = 0}^{m} \hat{f}\left( \xi \right), \mathop \sum \limits_{l = 0}^{m} \hat{g}\left( \xi \right), \mathop \sum \limits_{l = 0}^{m} \hat{\theta }\left( \xi \right),\mathop \sum \limits_{l = 0}^{m} \hat{\phi}\left( \xi \right)} \right)} \right]^{2} , $$

where

$$ e_{m}^{t} = e_{m}^{f} + e_{m}^{g} + e_{m}^{\theta } + e_{m}^{\phi } . $$

The optimal values at 3rd order are $H_{f} = - 1.125554, H_{g} = - 0.67757434, H_{\theta } = - 1.00771259, H_{\phi } = - 0.66544386,$ by fixing the involved parameters as $Sc = 0.5, Pr = 1.0, \alpha = 0.4, \beta_{1} = 0.5, \beta_{2} = 0.6, M = 0.1,\delta_{1} = 0.1, \delta_{2} = 0.3,\epsilon_{1} = 0.1,\epsilon_{2} = 0.2.$ Table 1 is prepared to notice the error analysis against higher order approximations.

**Table 1:** Computation of averaged squared residuals errors of velocity, temperature, and concentration solution.

Approximate Order (n)	$\delta_{n}^{f}$	$\delta_{n}^{g}$	$\delta_{n}^{\theta }$	$\delta_{n}^{\phi }$
2	$0.00004268$	$6.9894 \times 10^{ - 6}$	$0.00001112$	$0.0024046$
4	$1.09621 \times 10^{ - 6}$	$6.79463 \times 10^{ - 7}$	$1.23295 \times 10^{ - 6}$	$0.0003176$
8	$6.94405 \times 10^{ - 10}$	$1.68247 \times 10^{ - 8}$	$1.71512 \times 10^{ - 8}$	$0.00003448$
12	$1.45543 \times 10^{ - 11}$	$8.02786 \times 10^{ - 10}$	$6.00118 \times 10^{ - 10}$	$6.61207 \times 10^{ - 6}$
16	$1.73202 \times 10^{ - 13}$	$2.49836 \times 10^{ - 11}$	$1.87111 \times 10^{ - 11}$	$1.60319 \times 10^{ - 7}$
20	$9.10536 \times 10^{ - 15}$	$1.01889 \times 10^{ - 12}$	$1.3158 \times 10^{ - 13}$	$4.48226 \times 10^{ - 7}$

## Discussion and analysis on graphical and tabular results

The 3D flow situation of Prandtl fluid subjected to magnetic field under the action of Cattaneo–Christov heat flux is performed past a stretched surface. The variable properties called temperature dependent concentration and thermal conductivity are involved via slip conditions. This physical complex model is numerically simulated by numerical approach. The graphical simulations of heat energy, concentration and motion of fluid particles are conducted through graphs and tables while these simulations are given below:

### Simulations of flow phenomenon versus physical parameters

**Figure 2:**
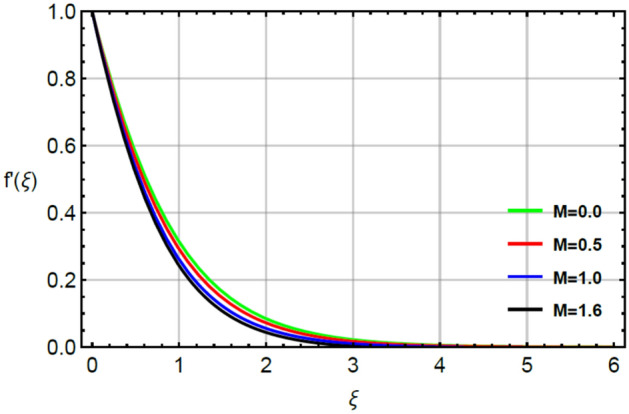
Character of $M$ on $f^{\prime}$.

**Figure 3:**
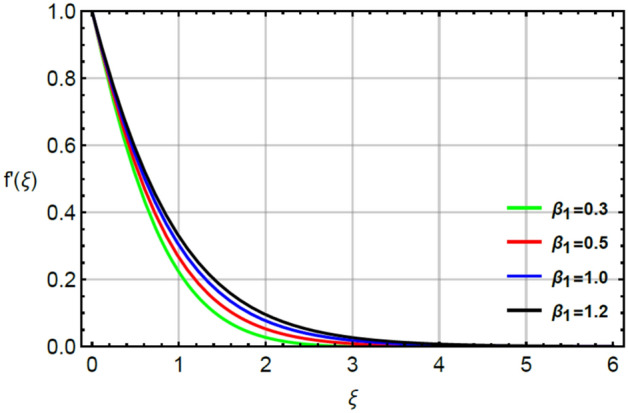
Character of $\beta_{1}$ on $f^{\prime}$.

**Figure 4:**
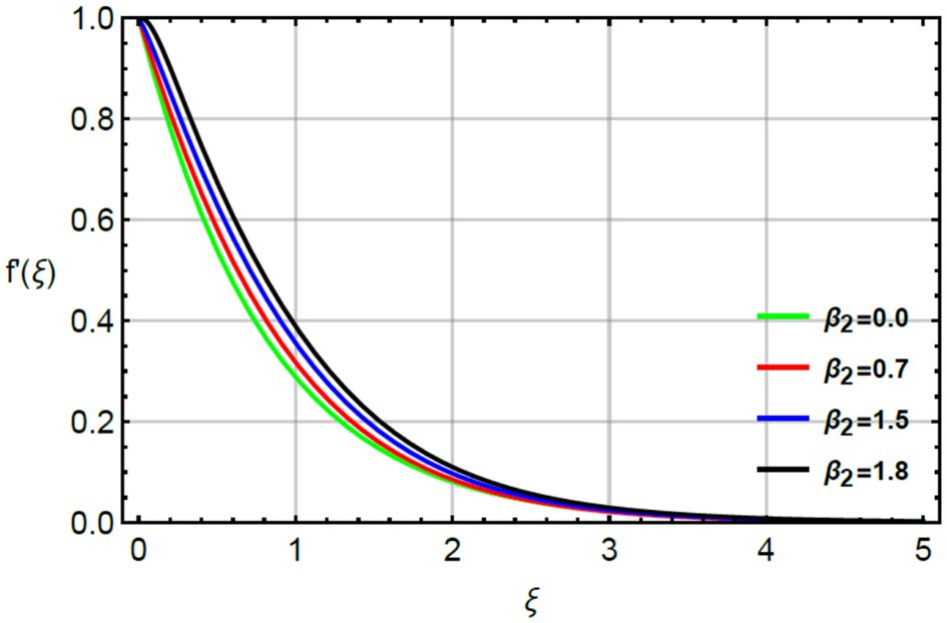
Character of $\beta_{2}$ on $f^{\prime}$.

**Figure 5:**
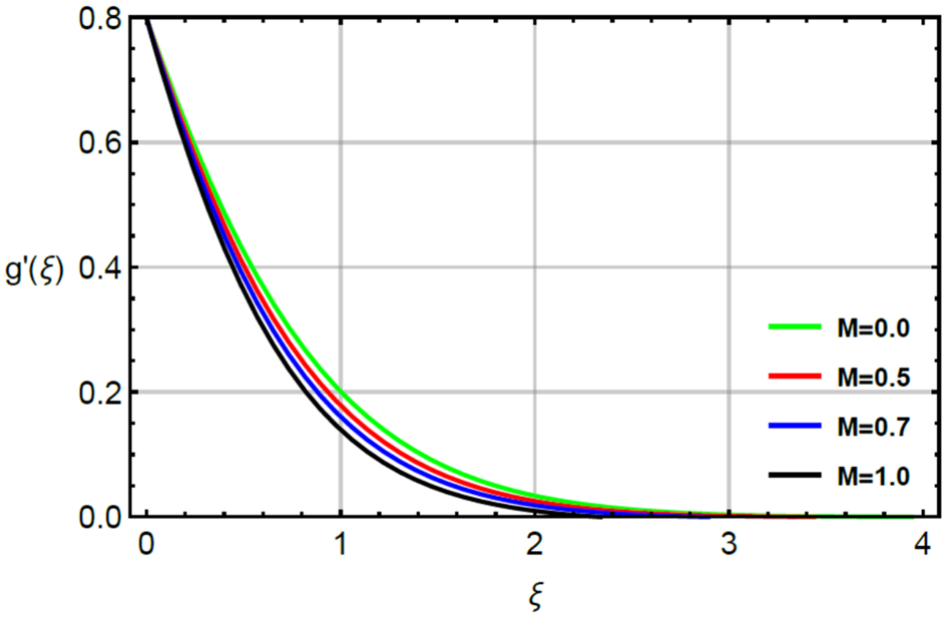
Character of $M$ on $g^{\prime}$.

**Figure 6:**
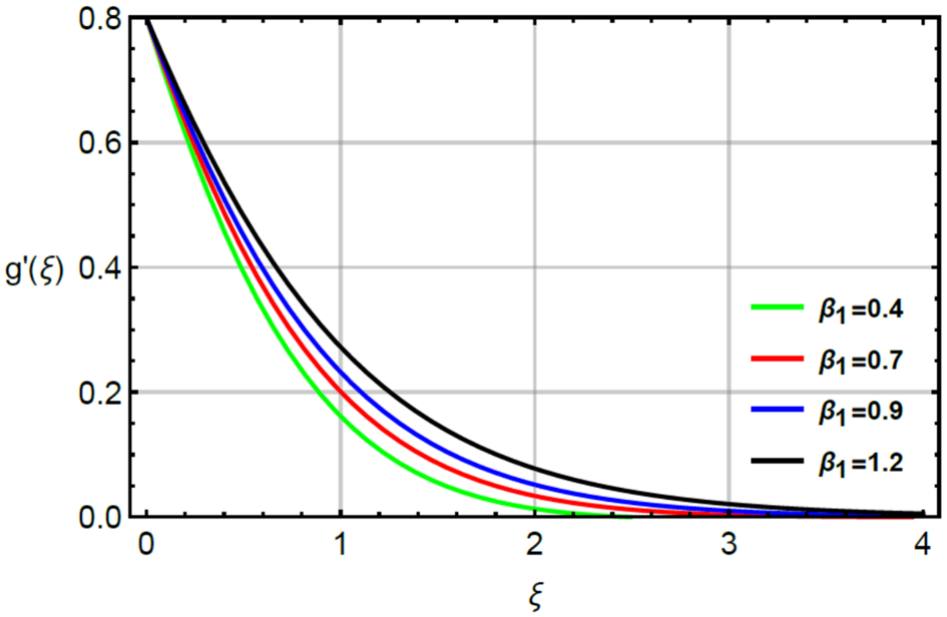
Character of $\beta_{1}$ on $g^{\prime}$.

**Figure 7:**
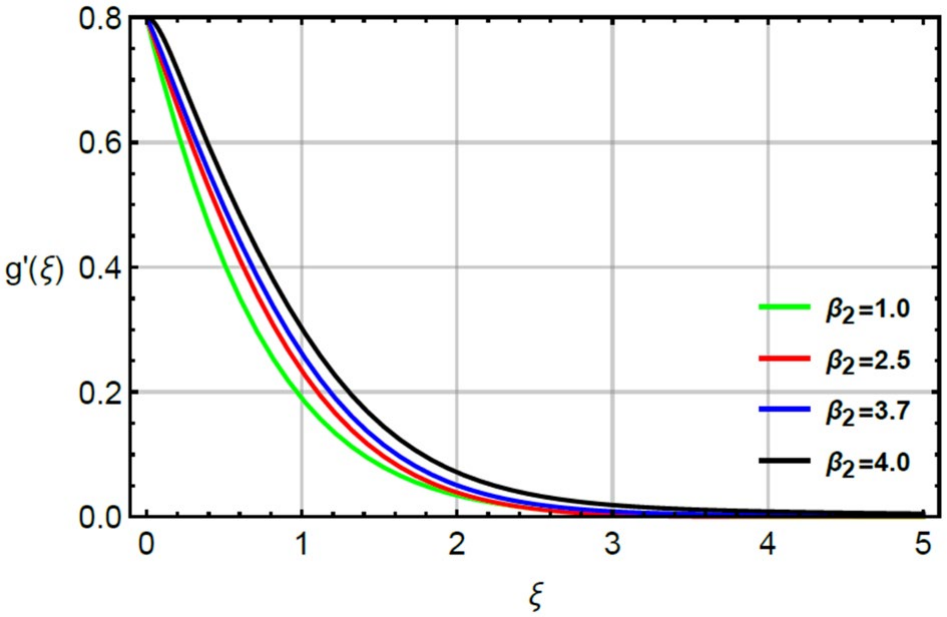
Character of $\beta_{2}$ on $g^{\prime}$.

The parameter called magnetic number captures features of Prandtl liquid under the action of non-Fourier’s model using variable properties in the existence of mass and heat transport. The phenomenal role of $M$ on x-and y-directions of velocities are illustrated by Figs. 2 and 5. The retardation force is generated in motion of fluid particles due to input of $M$ and this retardation makes slow in speed of fluid particles. Actually, the action of magnetic field generates frictional force called opposing force (Lorentz force) in Eqs. (12) and 13. The Lorentz forces in y- and x-directions of momentum equations are $- Mf^{\prime}\left( \xi \right)$ and $Mg^{\prime}\left( \xi \right)$ in Eqs. (12) and (13) respectively. Moreover, constant strength of magnetic field is taken along vertical of heated surface. The direction of magnetic field is applied against the direction of flow and this happening creates resistance during the flow of fluid particles. Hence, Lorentz forces are negative force which is opposite to direction of flow. Therefore, secondary and primary velocities become slow down due to large integration of magnetic number. The representation of $\beta_{1}$ called Prandtl fluid parameter on secondary and primary velocities is delineated by Figs. 3 and 6. The parameter ($\beta_{1}$) is appeared in the current model due to tensor of Prandt fluid. The direct relation is found between the flow of fluid particles and $\beta_{1} .$ An increment in $\beta_{1}$ brings the enhancement in motion of fluid particles. Hence, velocities are increased versus the large values of $\beta_{1} .$ Figures 4 and 7 portray the character of elastic parameter on the primary and secondary flows. To obtain the desired results of $\beta_{2}$, the range of $0.4 \le \beta_{2} \le 1.8$ is considered. It can be found that for enhancing the values $\beta_{2}$ results profiles of velocities in y-and x-directions are enhanced. This type of situation is validated for large values of $\beta_{2}$ makes increment in viscosity of fluid. By the definition of elastic parameter, it has direct relation against the dynamic viscosity of fluid. The viscosity of fluid is reduced when elastic parameter is increased. Hence, the role of elastic parameter accelerates the motion in fluid particles.

**Figure 8:**
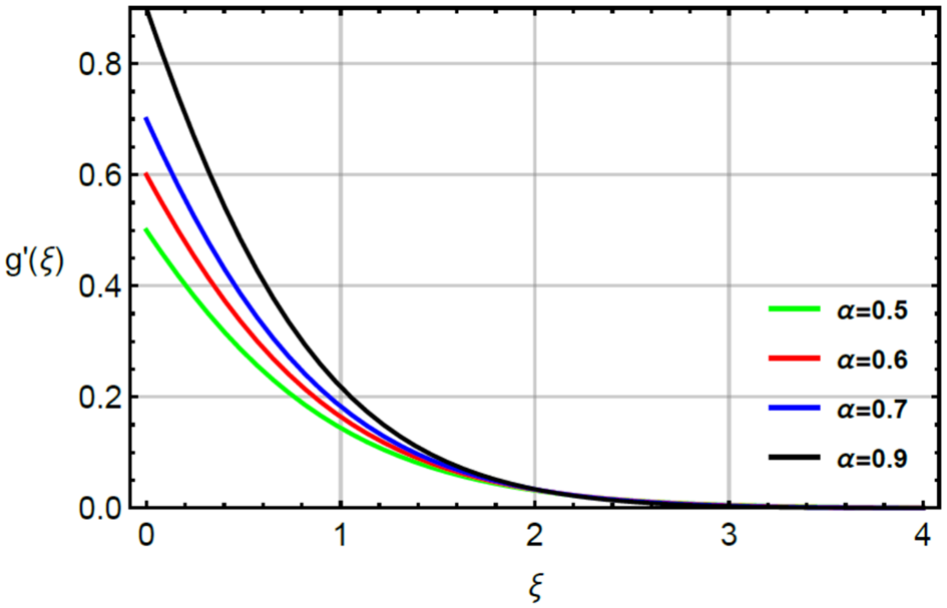
Character of $\alpha$ on $g^{\prime}$.

Figure 8 plots the role $\alpha$ on $g^{\prime}.$ It is estimated that from outcome sketch flow of fluid particles in y-and x-directions become enhanced by large values of $\alpha$. Moreover, viscosity of fluid becomes decrease by applying the increment of $\alpha$. So, less viscosity is generated for large values of $\alpha$ and secondary and primary velocities are enhanced. The ratio number is considered as ratio of horizontal movement and vertical movement. The motion of fluid particles is acerbated due to motion of elastic heated surface. Meanwhile, ration parameter plays a vital impact to maximize the motion in fluid particles.

### Simulations of heat energy versus physical parameters

**Figure 9:**
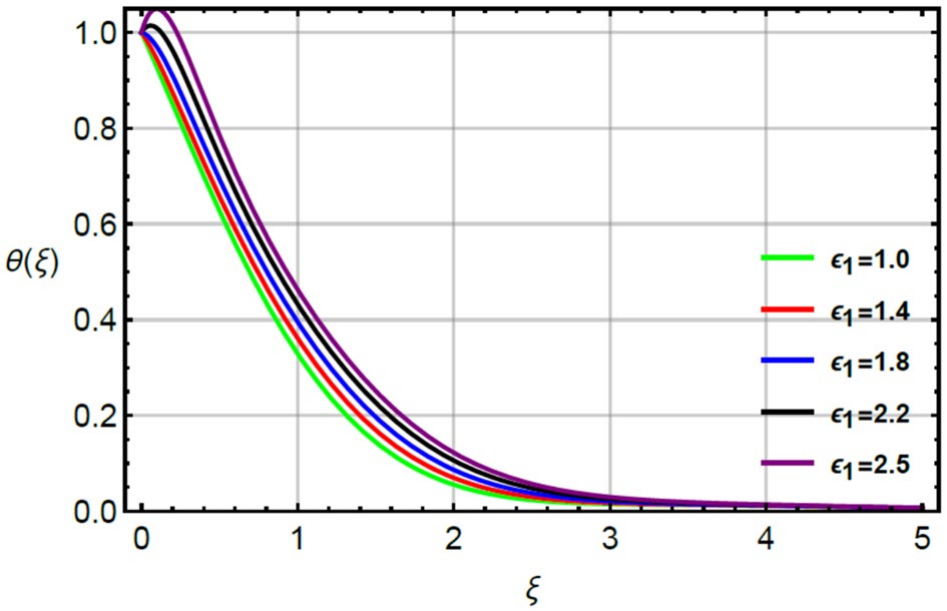
Character of $\epsilon_{1}$ on $\theta$.

**Figure 10:**
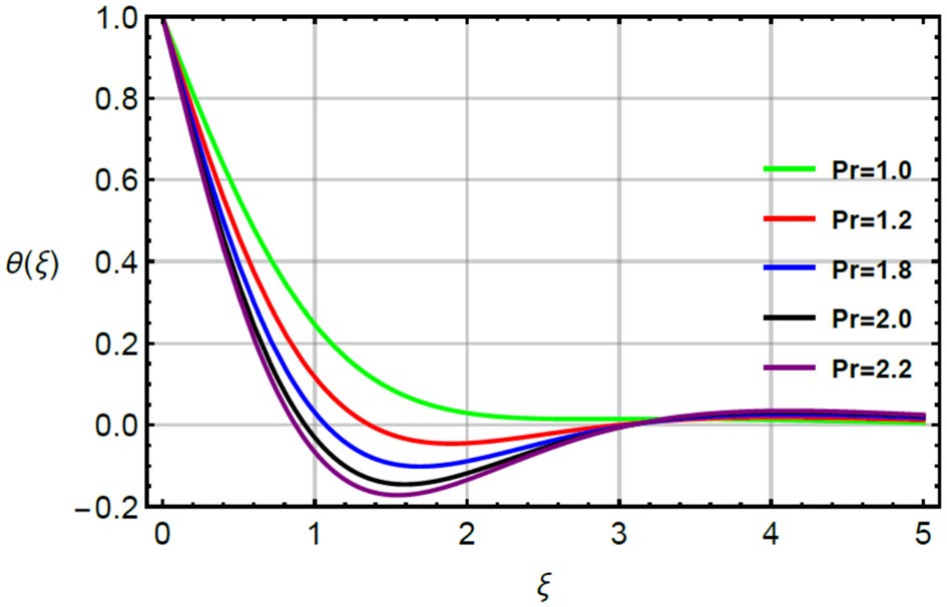
Character of $Pr$ on $\theta$.

**Figure 11:**
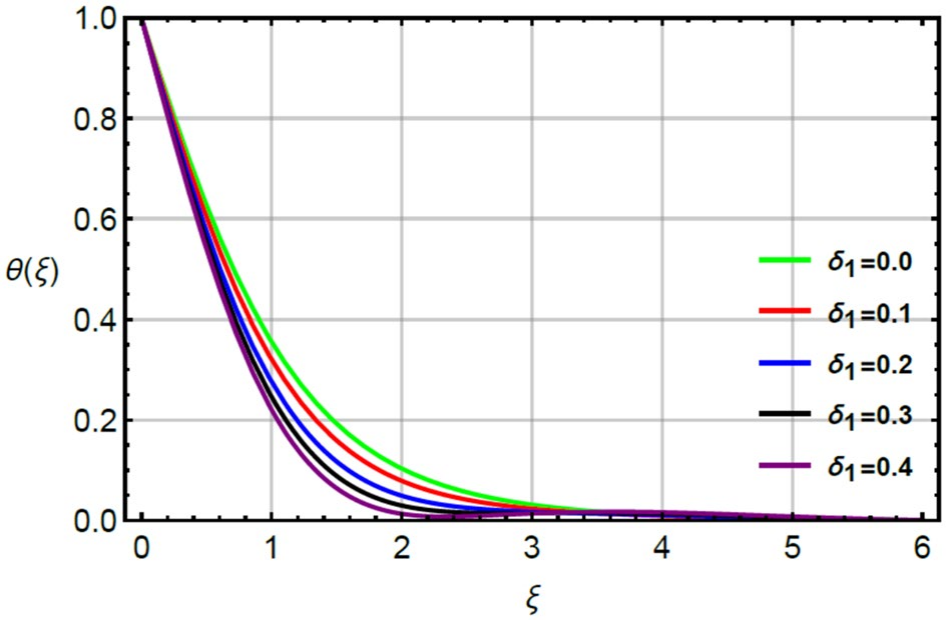
Character of $\delta_{1}$ on $\theta$.

The simulations of thermal energy are captured by variation of parameters called Prandtl $\left( {Pr} \right)$, very small $\left( {\epsilon_{1} } \right)$ and thermal relaxation $\left( {\delta_{1} } \right)$ by Figs. 9, 10 and 11. Figure 9 captures the variation in thermal energy with applying the large values of $\epsilon_{1}$. It is estimated that $\epsilon_{1}$ is appeared due to role of changing of thermal conductivity with respect to temperature in dimensionless energy equation while the appearance of $\epsilon_{1}$ decides the role of constant and variable thermal conductivities. From the Fig. 9, it can be clearly seen that enhancement in thermal energy is investigated versus large values of $\epsilon_{1}$. This enhancement in thermal energy is happened because $\epsilon_{1}$ has direct relation versus temperature in temperature dependent thermal conductivity model. So, this direct relation generates maximum heat energy via large values of $\epsilon_{1}$. The parameter related to $\epsilon_{1}$ is occurred due to variable thermal conductivity in energy equation (dimensionless). This parameter treats as linear function in thermal energy and an enhancement in $\epsilon_{1}$ results increase the random motion of fluid particles due to temperature gradient. An increment in random motion of fluid particles correspondences increment in heat energy of fluid particles is happened. Hence, temperature enhances. Figure 10 indicates the characterization of thermal energy with higher values of $Pr$. As, $Pr$ discusses the dual behavior of boundary layers regarding moment and thermal layers during the flow of fluid at boundary of the sheet. Actually, inverse relation between thermal layer and $Pr$. So, layers regarding thermal energy becomes declined versus the large values of $Pr$. Hence, increment in $Pr$ results the reduction in thermal energy. Physically, Prandtl is considered as a fractional between the momentum boundary and thermal boundary layers. By the definition of $Pr$, layers regarding thermal are based on variation of $Pr$ whereas thickness of thermal layers are increased versus the enlargement in $Pr.$ The term regarding thermal relaxation parameter is developed using concept of non-Fourier’s law in energy equation as well as in concentration equation. The appearance of $\delta_{1}$ decides the role of non-Fourier’s and Fourier’s laws in present flow model while the appearance of $\delta_{1}$ is simulated by Fig. 11. From this figure, layers regarding thermal energy attain less temperature. Consequently, temperature decreases with respect to large values of $\delta_{1} .$ In physical view, the term related to relaxation means reappearance of system into equilibrium condition and every process of relaxation are considered as a relaxation time. The large values of $\delta_{1}$ reveals an enhancement in capability of Prandtl fluid to return the equilibrium state while this happening brings the minimizing variation in thermal state of Prandtl fluid.

### Simulations of concentration versus physical parameters

**Figure 12:**
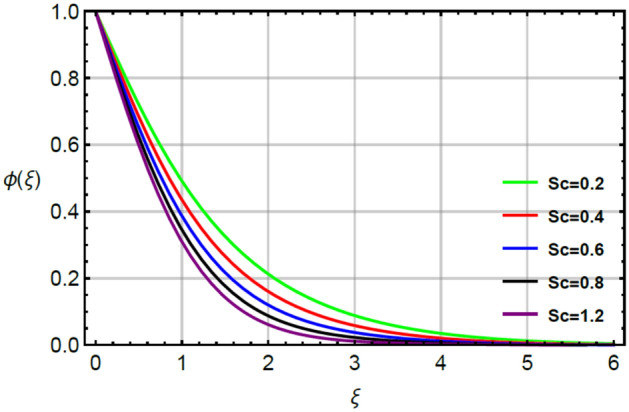
Character of $Sc$ on $\phi$.

**Figure 13:**
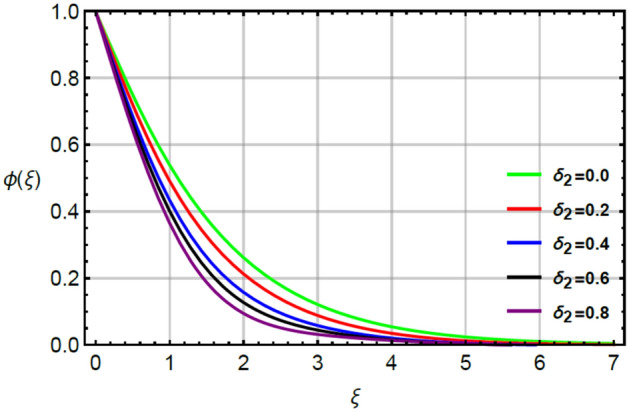
Character of $\delta_{2}$ on $\phi$.

**Figure 14:**
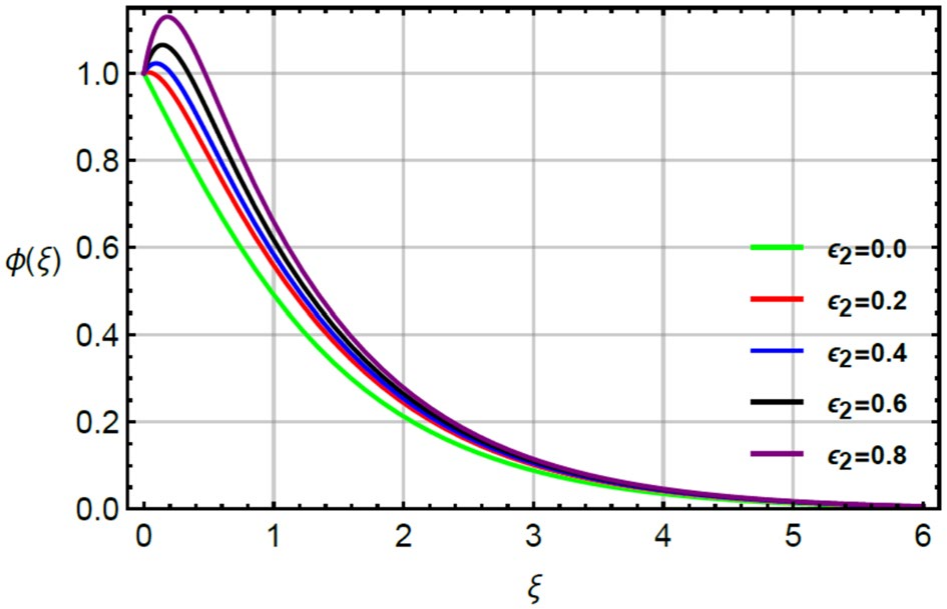
Character of $\epsilon_{2}$ on $\phi$.

Figures 12, 13 and 14 illustrate the influences of concentration with respect to variation of $Sc, \epsilon_{2}$ and $\delta_{2}$. Figure 12 conducts the role of $Sc$ on the curves of concentration. The division of mass and momentum diffusion coefficients is knows as $Sc$ while diffusion of mass particles becomes slow down. This decline in diffusion of particles is happened because of inverse relation between $Sc$ and mass diffusion coefficient. Physically, the solute particles are declined due the definition of $Sc.$ This decline in solute particles becomes slow down due to inclement in diffusion of particles. Hence, diffusion of fluid particles becomes slow down versus large values of $Sc.$ The variation in concentration field is investigated by $\delta_{2}$ while outcomes regarding concentration versus $\delta_{2}$ is displayed by Fig. 13. It is noticed that $\delta_{2}$ in dimensionless concentration equation is modeled due to the influence of non-Fourier’s law. The diffusion of solute particles for the case of (appearance of Cattaneo–Christov law) is higher than diffusion of solute particles for the case of (disappearance of Cattaneo–Christov law). So, the performance of concentration in the absence of $\delta_{2}$ is much better than for the case of non-Fourier’s law. So, higher values of $\delta_{2}$ make the reduction in profiles of concentration. It is noticed that $\delta_{2}$ has analog versus the Deborah number and the fluid has ability to return original state elated to concentration in the fluid. Figure 14 captures the character of $\epsilon_{2}$ on diffusion of fluid particles. The graph between the diffusion of fluid particles and $\epsilon_{2}$ is increasing. In this relation, diffusion of fluid particles becomes fast while $\epsilon_{2}$ is appeared due to variable concentration. Further, this increment in diffusion of fluid particles because $\epsilon_{2}$ exists as a direct relation versus concentration. So, this direct relation brings increment in diffusion of fluid particles (Figs. 2, 3, 4, 5, 6, 7, 8, 9, 10, 11, 12, 13, 14).

### Simulations of divergent velocities against physical parameters

The related simulations of divergent velocities versus the variation of $Pr$ and $\alpha$ are calculated by Tables 2 and 3. The improvement in divergent velocities in y-and x-directions versus the increment in Prandtl number is investigated. Hence, $\alpha$ makes great impact for the enhancement of shear stresses in y-and x-directions. It is also investigated that validation of numerical simulations is done through considered values $0.0, 0.25, 0.5, 0.75$ and $1.0$ of $\alpha$ with already published work of Wang^27^, Kumar et al.^28^ and Hayat et al.^29^. The remarkable comparison is found between present work and published results. Temperature gradient versus the various values of $Pr$ is captured by Table 3. From this table, temperature gradient is enhanced with respect to large values of $Pr$ whereas comparison of results are estimated by published work of Khan et al.^30^ and Bilal et al.^31^ by considering range of $Pr$ ($0.07 \le Pr \le 2.0$).

**Table 2:** Comparative analysis of dimensionless stress by fluctuating the values of velocity rates parameter and setting $M = 0.$

	Hayat et al.^29^		Wang^27^		Kumar et al.^28^		Present results	
$\alpha$	$- f^{\prime\prime}\left( 0 \right)$	$- g^{\prime\prime}\left( 0 \right)$	$- f^{\prime\prime}\left( 0 \right)$	$- g^{\prime\prime}\left( 0 \right)$	$- f^{\prime\prime}\left( 0 \right)$	$- g^{\prime\prime}\left( 0 \right)$	$- f^{\prime\prime}\left( 0 \right)$	$- g^{\prime\prime}\left( 0 \right)$
$0.0$	1.0	0.0	1.0	0.0	1.0	0.0	1.00000	0.000000
$0.25$	1.048810	0.19457	1.0488	0.1945	1.04906	0.19457	1.048124	0.194562
$0.5$	1.093095	0.465205	1.0930	0.4652	1.09324	0.46532	1.093054	0.465281
$0.75$	1.134500	0.794620	1.1344	0.7946	1.13458	0.79470	1.1345012	0.794624
$1.0$	1.173721	1.173721	1.1737	1.1737	1.17378	1.17378	1.173210	1.173210

**Table 3:** Computation of heat temperature rate for ($g = 0;$ i.e. two diemsional case) against Prandtl number by fixing other parameters.

$Pr$	Khan et al.^30^	Bilal et al.^31^	Present Results
0.07	0.06633	0.0660	0.066847
0.20	0.1691	0.1691	0.1697304
0.70	0.4539	0.5349	0.453721
2.00	0.9113	0.9114	0.911708

## Concluding key points

The numerical simulations of mass and heat energy using conservation laws under the action of non-Fourier’s law are numerically solved. The concept of variable properties in view of concentration and thermal conductivity in the presence of Prandtl fluid past a hot surface is considered. The outcomes against the physical parameters are concluded which are listed below:

- The magnetic field generates retardation force in motion of fluid particles and MBL (momentum boundary layers) become thick due to retardation force in y- and x-directions of the surface;
- The motion of fluid particles become fast in y- and x-directions of the hot surface by considering the enhancement in Prandtl, ratio and elastic parameters whereas thickness of momentum boundary layers are reduced using large values of Prandtl, ratio and elastic parameters;
- The less production of thermal energy is achieved versus variation in $Pr$ and $\delta_{1}$. The maximum heat energy is achieved for the case generalized Fourier’s law as compared for the case of non- Fourier’s law. Prandtl number makes vital impact for adjustment in TBL as well as in MBL;
- The maximum production of heat energy is obtained for the large values of $\epsilon_{1}$. Hence, $\epsilon_{1}$ plays a vital role for maximum production of heat energy;
- The diffusion of fluid particles becomes fast versus the enhancement of $\epsilon_{1}$. But diffusion of fluid particles becomes slow down versus large values of $Sc$ and $\delta_{2}$;
- The divergent velocities in y- and x-directions are decreased by the large values of ratio number and gradient temperature is enhanced due to variation in Prandtl number.
- The solution approach is called HAM analysis scheme is used to simulate the solution of present complex model while current analytical approach is very useful and it has many applications in industrial and engineering processes;
- The significant applications of current model are used in recovery of petroleum, enhancement of heat energy, food making process, energy devices and adjusting cooling devices etc.

## Data Availability

The data used to support this study are included in the article.
